# Mapping and enumerating houses and households to support malaria control interventions on Bioko Island

**DOI:** 10.1186/s12936-019-2920-x

**Published:** 2019-08-22

**Authors:** Guillermo A. García, Dianna E. B. Hergott, Wonder P. Phiri, Megan Perry, Jordan Smith, Jose Osa Osa Nfumu, Jeremías Nzamio, Godwin Fuseini, Thomas Stabler, Matilde Riloha Rivas, Immo Kleinschmidt, Christopher Schwabe, Carlos A. Guerra

**Affiliations:** 1 Medical Care Development International, Silver Spring, MD USA; 2 Department of Epidemiology, University of Washington, Seattle, WA USA; 3 Medical Care Development International, Malabo, Equatorial Guinea; 4 Sanaria Inc., Rockville, MD USA; 5 Equatorial Guinea Ministry of Health and Social Welfare, Malabo, Equatorial Guinea; 6 London School of Hygiene and Tropical Medicine, London, UK; 7 Medical Care Development, Inc., Augusta, ME USA

**Keywords:** Malaria, Household, Housing, Enumeration, Control interventions, Bioko Island

## Abstract

**Background:**

Housing mapping and household enumeration are essential for the planning, implementation, targeting, and monitoring of malaria control interventions. In many malaria endemic countries, control efforts are hindered by incomplete or non-existent housing cartography and household enumeration. This paper describes the development of a comprehensive mapping and enumeration system to support the Bioko Island Malaria Control Project (BIMCP).

**Results:**

A highly detailed database was developed to include every housing unit on Bioko Island and uniquely enumerate the associated households residing in these houses. First, the island was divided into a virtual, geo-dereferenced grid of 1 × 1 km sequentially numbered *map*-*areas,* each of which was in turn subdivided into one hundred, 100 × 100 m sequentially numbered *map*-*sectors*. Second, high-resolution satellite imagery was used to sequentially and uniquely identify all housing units within each *map*-*sector*. Third, where satellite imagery was not available, global positioning systems (GPS) were used as the basis for uniquely identifying and mapping housing units in a sequential manner. A total of 97,048 housing units were mapped by 2018, 56% of which were concentrated in just 5.2% of Bioko Island’s total mapped area. Of these housing units, 70.7% were occupied, thus representing uniquely identified households.

**Conclusions:**

The housing unit mapping and household enumeration system developed for Bioko Island enabled the BIMCP to more effectively plan, implement, target, and monitor malaria control interventions. Since 2014, the BIMCP has used the unique household identifiers to monitor all household-level interventions, including indoor residual spraying, long-lasting insecticide-treated nets distribution, and annual malaria indicator surveys. The coding system used to create the unique housing unit and household identifiers is highly intuitive and allows quick location of any house within the grid without a GPS. Its flexibility has permitted the BIMCP to easily take into account the rapid and substantial changes in housing infrastructure. Importantly, by utilizing this coding system, an unprecedented quantity and diversity of detailed, geo-referenced demographic and health data have been assembled that have proved highly relevant for informing decision-making both for malaria control and potentially for the wider public health agenda on Bioko Island.

## Background

Bioko Island is located in the Bight of Biafra, off the coast of Cameroon, and is the largest and most important island of Equatorial Guinea. Bioko Island has an area of approximately 2000 km^2^ and a population of about 250,000 inhabitants, 80% of whom live in the main city, Malabo, which is also the country capital. The Bioko Island Malaria Control Project (BIMCP) was established in 2003 as part of the National Malaria Control Programme (NMCP) of the Ministry of Health and Social Welfare of Equatorial Guinea to implement malaria control interventions with the aim of reducing the burden of, and ultimately eliminating, the disease on the island [1–5].

In 2018, the BIMCP completed its third 5-year phase, having achieved significant reductions in malaria transmission as measured by parasite prevalence, fever cases, anemia and all-cause mortality in young children [3, 6, 7]. The success also is reflected in entomological indicators, with significant reductions in anopheline human biting rates [8], sporozoite rates, and vector density [9]. Recent analyses strongly suggest that autochthonous transmission may have been interrupted in many areas of the island and that observed prevalence could be largely attributed to importation [10]. Behind these successful outcomes are years of funding that assured the scale-up of malaria interventions, including 10 years of extensive and intensive indoor residual spraying (IRS) followed by 5 years of targeted focal spraying, several mass distribution and hang-up campaigns for long-lasting insecticide-treated nets (LLINs) sustained by continuous distribution through antenatal clinics and intermittent primary-school campaigns, focal targeted larval source management, universally free malaria diagnosis and treatment, and intermittent preventive therapy for pregnant women. This high coverage of interventions has been monitored through entomological surveillance, annual malaria indicator surveys (MIS), and a health information system based on individual patient records [11, 12].

Effective resource allocation for malaria control depends on accurate denominators that define and fully characterize the relevant intervention universe [13]. Among the challenges faced by the BIMCP during the initial phase of the project was the limitation of operating without detailed housing unit cartography and household enumeration to support effective planning and implementation of interventions. Households were counted within communities of which there was limited spatial knowledge. The absence of a geo-referenced households database thus translated into uncertain and inaccurate knowledge of the true denominator of units for intervention and, therefore, into sub-optimal planning, targeting and coverage monitoring [1]. The impact of these limitations was further exacerbated by the rapid growth in housing stock associated with the booming oil-driven economy, including substantial growth in informal urban housing in Malabo.

Accurate household enumeration, therefore, is key for the implementation of household-based interventions. Moreover, there is an increasing need for scaling-up or adapting malaria control and much of this can be informed by wide-scale household surveys [14]. Notwithstanding, for many malaria control programmes, enumeration methods remain rudimentary or nonexistent and relatively little effort seems to have been invested in instituting mapping and enumeration systems as part of malaria control programmes. Recently, new methodologies have been tested driven by the need to reassess vector control strategies towards more targeted approaches in sub-Saharan malaria endemic areas [15, 16]. In Mozambique, houses in Mopeia district were enumerated by the use of GPS and satellite imagery triangulation; satellite images allowed the identification of areas that had been missed in the first round of enumeration that later were revisited for inclusion. In Zambia, open-source satellite imagery was used for household enumeration in 15 districts and proposed as a more efficient alternative to field enumeration methods. This methodology was tested in rural areas where housing density was low and atmospheric conditions proved favourable (i.e. limited cloud cover), and was used later for targeting IRS activities [17].

This paper describes the experience developing a cartographic system to map housing units and enumerate households on Bioko Island and how it has enabled the BIMCP to substantially improve its planning, service targeting, impact and coverage monitoring, to enhance the effectiveness of its interventions and to ultimately achieve a continued reduction of malaria burden on the island. The system was developed with the approval and collaboration of the NMCP. For clarity, the paper refers to buildings destined for human habitation as houses or housing units, and as households to groups of people that reside in a housing unit and who regularly share meals together. Therefore, housing units may be occupied (have a resident household) or unoccupied (have no resident household).

## Methods

The process of mapping housing units and enumerating households was initiated on a pilot basis in one community in 2011, extended to 18 sentinel sites [2] later in the same year, and systematically implemented on the whole island by 2014. Bespoke application software was used to support enumeration and data entry in the field, at first with the use of electronic personal digital assistants (PDAs) and later through the use of tablet computers. The methodology involved developing a consistent coding system for uniquely identifying and mapping housing units with the aid of remote sensing imagery and global positioning systems (GPS), as well as geo-referencing them through an interaction between teams collecting data in the field (i.e. ground truth of locations) and data analysts assembling the information on a geographical information system (GIS). The ultimate aim of this process was to have a complete geographical database of housing units, each assigned a unique identifier and linked to several attributes, including housing occupancy and household information. This methodology is described in more detail below.

### A system for uniquely identifying housing units

**Fig. 1:**
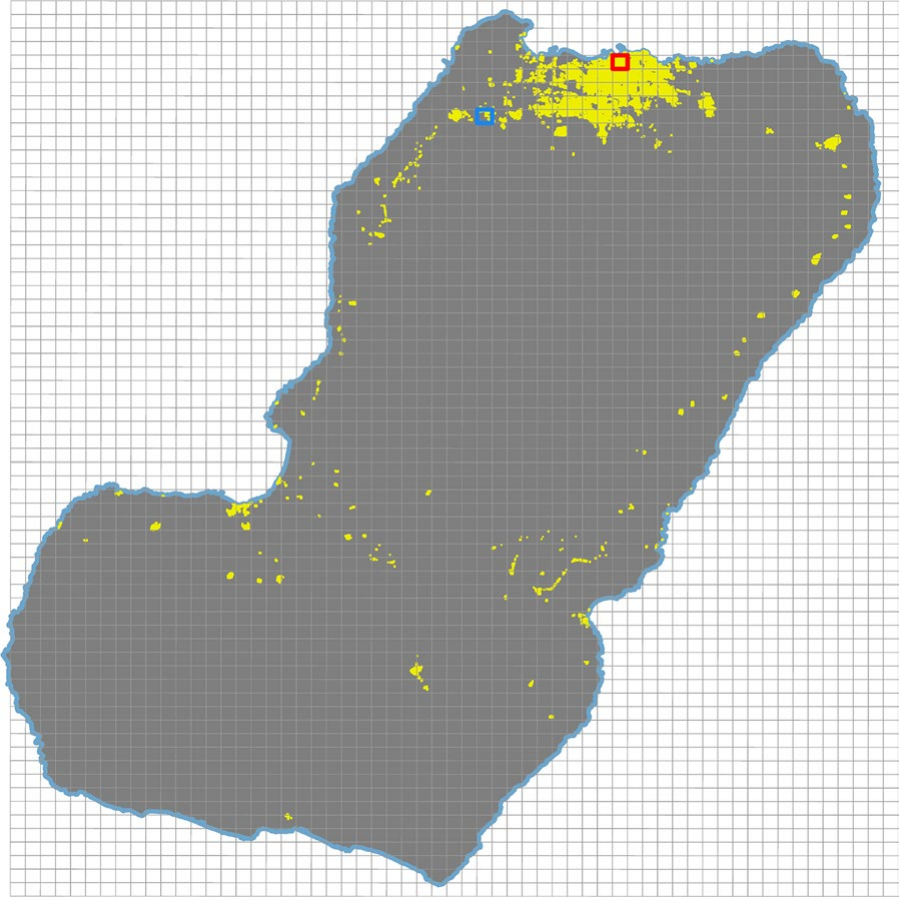
The mapping and coding system for Bioko. A virtual grid of 1 × 1 km *map*-*areas* (grey lines) was used to generate unique identifiers. Example *map*-*areas* M0277 (urban) and M0504 (rural) are highlighted with red and blue boxes, respectively. The yellow dots represent housing units in 2018; grey areas are uninhabited

**Fig. 2:**
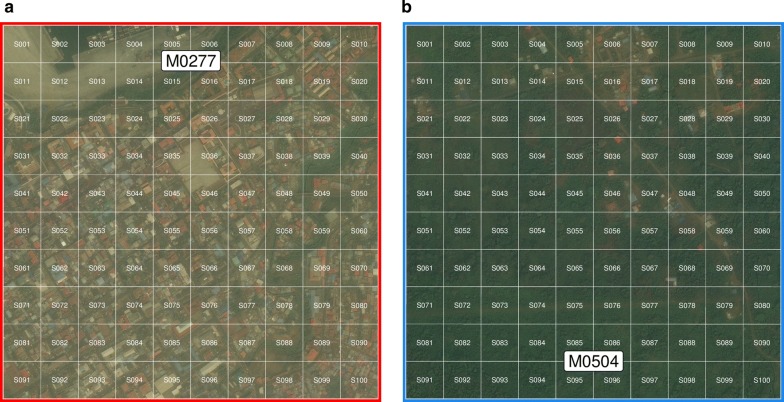
Detail of *map*-*areas* M0277 (urban) and M0504 (rural). A grid of 100 × 100 m *map*-*sectors* (white lines) subdivided each *map*-*area* into 100 cells. The satellite images shown here were sourced from Digital Globe [18]

A coding system was established by first creating a virtual, rectangular, geo-referenced grid of 59 by 66 1 × 1 km cells on a GIS that covered the whole territory of Bioko Island (Fig. 1). These cells, referred to as *map*-*areas*, were sequentially numbered from left to right and top to bottom, that is from cell 0001, at the top left, to cell 3894, at the bottom right of the grid. Another grid divided each *map*-*area* into one hundred, 100 × 100 m cells, referred to as *map*-*sectors* (Fig. 2). *Map*-*sectors* were numbered within each *map*-*area* in the same way, from left to right and top to bottom, from cell 001 to cell 100.

**Fig. 3:**
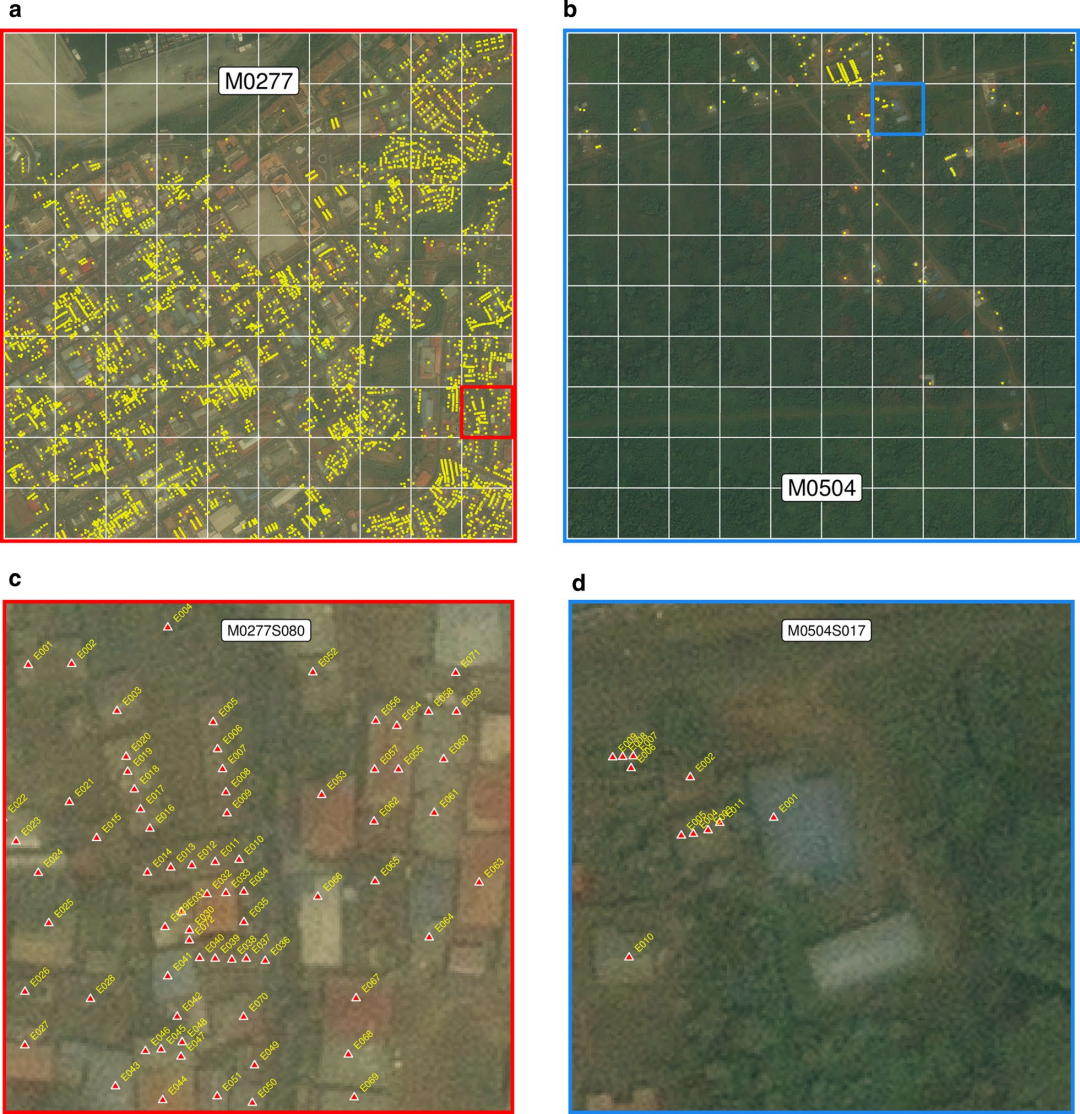
Location of mapped housing units. **a**, **b** Yellow dots illustrate housing units within *map*-*areas* M0277 (urban) and M0504 (rural) (Fig. 1). **c**, **d** Housing units and their unique identifiers within *map*-*sectors* M0277S080 and M0504S017 (red and blue boxes in **a** and **b**). Note that buildings without unique identifiers do not correspond to housing units. The satellite images shown here were sourced from Digital Globe [18]

Unique identifiers for each *map*-*area* and each *map*-*sector* were represented by their number preceded by a letter M, for *map*-*area*, and a letter S, for *map*-*sector*. That is, a letter M followed by four digits (between 0001 and 3894) uniquely identified *map*-*areas* and a letter S and three digits (between 001 and 100) uniquely identified *map*-*sectors*. For example, code M0277S080 is the unique identifier for *map*-*sector* 080 within *map*-*area* 0277 (Fig. 2). Unique identifiers for housing units within *map*-*sectors* were assigned using a letter E (in reference to the word “edificio”, or building in Spanish) followed by three digits. Hence, the code M0277S080E011 uniquely identifies the 11th housing unit found within sector M0277S080 (Fig. 3). Multiple housing units corresponding to apartments on different floors within the same building were assigned different unique identifiers. In addition, a letter P (standing for “piso”, or floor/level in Spanish) followed by two digits defined the floor where the housing unit was located (i.e. using the same example above, the full code for the housing unit, if it were located on the second floor, would be M0277S080E011P02).

### Mapping housing units

High-resolution satellite imagery was available between 2011 and 2014 only for the *map*-*sectors* in the Malabo area and surroundings [18]. In these cases, mapping teams were provided with printed maps containing the satellite image and the limits of the *map*-*sector*. In *map*-*sectors* for which satellite imagery was unavailable, or where such imagery was of a poor resolution or was cloud-obscured, the teams used the *map*-*sector* limits overlaid on detailed hand-drawn maps of streets, roads and pathways produced by the BIMCP using GPS, where they drew in buildings, locating these in space relative to these features. This was the case for most of the areas outside of Malabo, including rural and peri-urban areas. However, as updated, extended and improved resolution satellite imagery became progressively available over time, *map*-*sector* maps were converted from the GPS-coordinate dependent hand-drawn maps to satellite-based maps.

Mapping teams walked each *map*-*sector* on a path pre-determined on the printed maps, usually following a “serpentine pattern” starting in one corner and ending in the opposite corner of the *map*-*sector*. Mappers drew the outline of each building on the satellite image, or sketched out any new ones that they found. For *map*-*sectors* with no satellite imagery, they sketched out the buildings on the printed map and also took GPS readings. All housing units, whether they were occupied by households or not, were numbered sequentially in the order they were found along the predetermined path. In the case of housing units falling on the limits and overlapping two *map*-*sectors*, a decision was made to assign them to one or the other. The number for each housing unit (i.e. the three digits that follow the letter E in the unique identifier) was generated automatically by the PDA/tablet application and this was written down within or near the building outline on the paper map. For each housing unit, additional information was gathered such as a brief description of the house for easier identification in future visits (e.g. physical appearance, nearby landmarks), construction materials (e.g. wood, cement), other housing characteristics (e.g. open eaves, screened windows/doors, air-conditioning units) and, for occupied housing units, the demographics of all household members. A sticker with the unique identifier was affixed to the entrance door of each housing unit.

All printed maps sketched by the field teams were scanned to manually assign geographical coordinates to housing units based on the satellite imagery. For *map*-*areas* with no underlying images, the GPS coordinates obtained in the field were used. The geographical coordinates were linked to the data gathered on the PDA/tablet application and this completed the housing unit records.

## Results

**Fig. 4:**
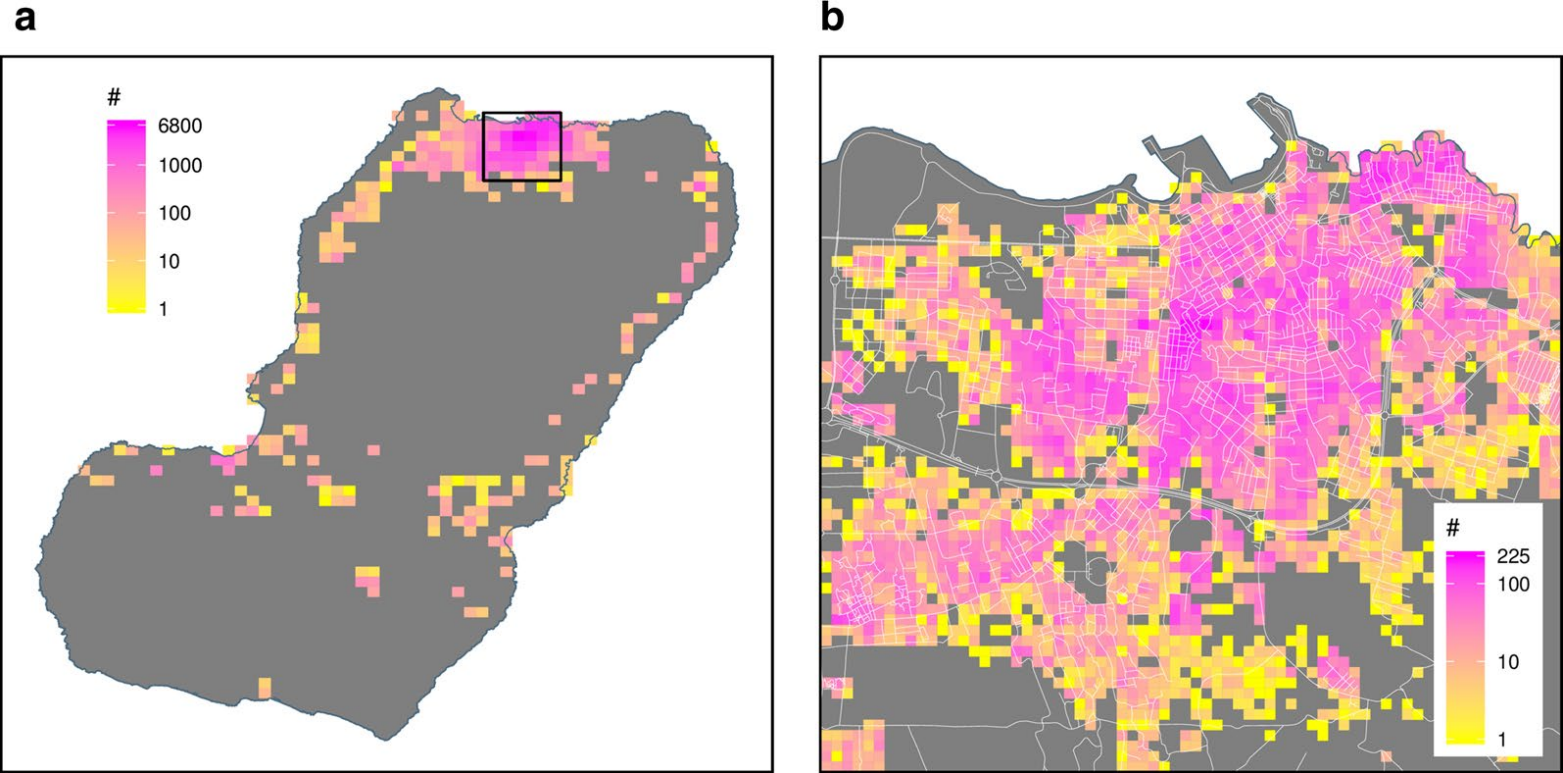
Housing unit density on Bioko Island. Density is shown by **a** *map*-*area* and **b** *map*-*sector.* The black box in **a** delimits the detail of Malabo shown in **b**

By the close of the first phase of the mapping of housing units and enumeration of associated households in 2014, 78,524 housing units had been mapped. Due to the rapid increase in housing infrastructure over time, by mid-2018, 18,524 new housing units had been added to the database to total 97,048 houses within 251 *map*-*areas* and 4467 *map*-*sectors* (Fig. 1), of which 68,619 (70.7%) were occupied. This represented an overall increase of 23.6% in the housing stock since the baseline mapping was completed, or an average 5.9% increase per annum (Table 1). Figure 3 illustrates mapped housing units in a high population density urban setting and in a low population density rural setting together with their unique identifiers. In these examples, there were 4373 mapped housing units in urban *map*-*area* M0277 compared to 164 in rural M0504, and 73 and 11 mapped houses in *map*-*sectors* M0277S080 and M0504S017, respectively. Housing density by *map*-*area* was highly skewed, ranging from 1 to 6840 housing units, with a median of 52 (Fig. 4). Fifty six percent of mapped housing units were concentrated in only 13 (5.2%) *map*-*areas*. The city of Malabo accounted for 83.8% of all houses on the island, with a median number of housing units per *map*-*area* of 707. House density at the *map*-*sector* level ranged between 1 and 227, with a median of 10. Over one quarter (26%) of mapped housing units were contained within 5.1% of *map*-*sectors* on Bioko.

**Table 1:** Number of mapped housing units, by year

Year	# houses	% increase
2014	78,524	–
2015	82,567	5.1
2016	89,023	13.4
2017	95,963	22.2
2018	97,048	23.6

The percent of increase is relative to the baseline number in 2014

Table 2 summarizes the benefits of having a geo-referenced housing/household database for implementing key interventions and surveillance on Bioko Island (i.e. IRS, LLIN distribution and annual MIS). These were benefits at the household-level and at the community-level relative to implementation of malaria control activities before the household database was established. Because individual household tagged with unique identifiers could be followed-up longitudinally, the programme could verify the continuity of interventions and of survey information. Based on these data, households could be individually targeted during, for example, IRS rounds. At community-level, household information provided the denominators upon which to plan interventions and quantify the resources needed to reach coverage targets. These denominators were also the basis for assessing intervention coverage, something that was not possible before the introduction of the household database.

**Table 2:** Benefits of a geo-referenced household database for implementing key interventions on Bioko Island

Intervention	Benefits
IRS/LLINs	*Household*-*level* Ability to track household interventions longitudinally based on permanent and unique household identifiers (e.g. track intra-household LLIN movement, monitoring insecticide concentrations following spray rounds) Ability to target households during initial and follow-up rounds of interventions Ability to verify and track sprayed households Ability to target interventions based on household-level data *Community*-*level* Ability to adequately quantify insecticide and LLIN requirements Improved deployment based on geo-political boundaries and known confirmed inhabited households Improved field supervision of control teams, including control of the true coverage achieved Ability to adequately engage community leaders to support interventions, particularly in urban areas
Household surveys (MIS)	*Household*-*level* Ability to link surveyed households longitudinally Ability to easily locate and survey pre-selected households *Community*-*level* Improved supervision to track and verify surveyed households Ability to estimate malaria prevalence at different spatial scales

## Discussion

The mapping and enumeration system developed for Bioko proved to be instrumental support for malaria control interventions on the island. The ability to uniquely identify and locate housing units and households substantially enhanced the planning, managing and monitoring of malaria control interventions and the accuracy with which service coverage was measured. Since 2014 this has been done through the Campaign Information Management System (CIMS), an Android-based application built around the household database and used as a tool for identifying and locating households targeted for interventions and for surveying. The household database provides the denominator for managing interventions, monitoring coverage and setting desired targets, particularly for IRS and LLINs distribution campaigns. Household level interventions can be monitored and followed longitudinally. As such, it is possible to know how many times each house was sprayed, received LLINs and was surveyed within any given time frame. Improved control of intervention teams is achieved when comparing outcomes against known denominators. Moreover, as information is uploaded to the CIMS, real-time monitoring and quality assurance of interventions is possible. All data entered through the CIMS, including current and new housing and household data, are owned by the NMCP. The data are encrypted and hosted on an online server and are accessible only to authorized users.

Demographic and environmental conditions on Bioko Island can limit the application of satellite imagery alone for mapping households, as was proposed by Kamanga and colleagues in their study in Zambia [16]. First, high population density urban areas on Bioko are equally targeted for control but difficult to map using remote sensing images. Second, open-source satellite imagery is usually outdated by between 1 and 3 years and, without complementary field-based validation methods, becomes quickly obsolete in the face of a rapid rate of housing growth like the one observed on Bioko Island. Third, cloud cover over the island can frequently hamper the use of remote sensing data. Finally, in both urban and rural areas of Bioko, many families live under the same roof. This general cultural household configuration is complicated by the fact that in rural areas households are relatively close to each other. It is impossible to tell that several families live under the same roof without field verification of satellite imagery. Hence, the mapping methodology used a hybrid approach that resorted to satellite imagery, GPS mapping and ground verification. In this way, it was possible to optimize the advantages provided by each method whilst overcoming their inherent limitations given by the specific context of Bioko Island. As a result of this exercise, all households on the island have been verified on the ground for accuracy to confirm that they exist, providing high confidence in the household counts.

Mapping housing units and enumerating households was particularly challenging and labor intensive in areas containing thousands of housing units per square kilometre. The system was developed on a learning-by-doing basis that increased the man-power needed to complete the mapping by, for example, assigning teams specifically for this task. One valuable lesson that was learned as the mapping progressed was that the mapping can be done at the same time and by the same personnel carrying out field interventions. This will not only reduce the burden of hours required to complete the mapping but also presents no limitation on how big or small an area can be mapped. It also makes the maintenance and updating of the household database relatively simple and straightforward during field visits by survey, spraying, and net distribution teams. One approach could be to use remote sensing to provide baseline count of buildings in a particular area in order to provide an a priori estimate of the resources needed to undertake household mapping and carry out an intervention. In such a scenario, the field team would verify the pre-identified buildings and then would assign codes to the households identified within those buildings. Regardless of the initial methodology adopted for the baseline mapping, the grid-based coding system allows quick allocation of household codes during visits without necessarily using satellite imagery. *Map*-*areas* and *map*-*sectors* provide unambiguous, geo-referenced boundaries where households can be mapped and coded, even without the use of GPS.

The methodology presented here was appropriate in a context like that of Bioko Island, which is similar to many malaria endemic settings, where urban areas lack a postal code system, so the virtual grids provided a simple, location-based way to assign unique identifiers. Because *map*-*areas*, *map*-*sectors* and houses are numbered sequentially, it is intuitive to navigate within the grids and locate houses based simply on unique identifiers. This has proven a big advantage particularly in the high-density housing areas of Malabo. In some way, the grid-based system described resembles a similarly innovative, location-based coding system that was recently developed to enumerate households in a densely populated impoverished area of Mumbai, India. Based on a virtual coordinate grid, this system allowed quick location of houses where the utility of GPS and satellite images proved limited [19]. The grid-based coding system also allows for rapid updating as new housing develops and households move between housing units. Because mapping activities have been integrated into all household-based interventions, field teams are trained to update the mapping system in real-time; in this way, new housing units are assigned the next subsequent code available. Given the close to 6% annual increase in housing infrastructure and the 30% unoccupancy of housing units on Bioko, which are suggestive of a high household turnover, this proved a particularly valuable feature of the mapping system.

The grid-based system provides further benefits. Since it was established, all data collected on Bioko Island at the household or individual level can be readily geo-referenced by indexing on the unique household identifier. Data can then be analyzed at various levels of aggregation, from individual, to household, to *map*-*sector*, to *map*-*area* level. The medical intelligence assembled by the BIMCP has thus reached a high level of sophistication. While the virtual grid system has no relevance from the standpoint of demarcating administrative boundaries, from a scientific and programmatic perspective it has the benefit of allowing analyses to be made within coherent spatial scales unaffected by the potential arbitrariness of political boundaries. For example, the grid-based system was used as the basis for the design of a cluster-randomized trial comparing the effectiveness of insecticides used for IRS in central Malabo [20]. More recently, *map*-*area*-based analyses were used to investigate human mobility and malaria prevalence in relation to malaria importation to Bioko [10].

The impact that the mapping system and the household database have had overall on malaria control activities on Bioko Island is wide-ranging (Table 2). The BIMCP has conducted four MIS, five IRS rounds and two LLINs mass distribution campaigns since the household database was established. This has allowed tracking the impact of interventions not only at the household level but also over time. The LLINs mass distribution campaigns of 2015 and 2018 achieved 88% and 85% coverage, respectively, and those households who did not receive LLINs, and the reasons for this, were recorded in the database. Pilot studies to assess the quality of LLINs have been possible by randomly selecting households that received nets to check for physical condition and quantify insecticide levels. Similarly, IRS coverage assessment has been possible with a high degree of accuracy and the household database has allowed recording why some houses were not sprayed. Plans are also underway to implement IRS targeting at the *map*-*sector* level; this increase in granularity of intervention planning will allow better targeting of transmission hot spots and improved assessment of intervention coverage. The household database also supports quality control of spraying rounds whereby sprayed houses are randomized for wall sample collections to quantify insecticide levels. Because sprayers are linked to the households they sprayed, based on this quality control, sprayers are retrained to improve their skills for subsequent rounds. Entomological surveys have also recently been incorporated to the CIMS to improve on the information derived from entomological monitoring. The household database is useful for recruiting households and individuals for ongoing and future studies. These include a malaria incidence study that is underway in a high prevalence area, as well as future vaccine trials on Bioko Island to assess the efficacy of a malaria vaccine candidate [21].

During the mass LLINs distribution campaigns, two population censuses were conducted that linked individuals to their household identifiers [22, 23] and these have provided a very high spatial resolution rendering of human population distribution on the island. These census data can be aggregated to the *map*-*sector* or *map*-*area* level for stratified, household sampling as one would do using gridded population surfaces [24]. Importantly, given its high spatial resolution, the *map*-*sector* grid is less prone to modifiable area unit problems that are evident when using large spatial units [24]. Therefore, *map*-*areas* and *map*-*sectors* can be effectively used as primary and secondary sampling units for multi-stage sampling surveys for a number of different purposes [14, 24, 25]. The household database could also provide the infrastructure for establishing a demographic surveillance system on Bioko Island [26]. More work is needed to analyze the extensive data sets assembled by the BIMCP at various spatial scales, to fully utilize the household database and mapping system and to assess their potential for numerous applications beyond malaria control. Though the mapping system was developed specifically to guide malaria control interventions, the household data are planned to support other public health interventions in the future and have already been used to inform poliomyelitis vaccination campaigns. In addition, the household database is being used by other governmental offices for the implementation of household-based surveys of various natures. Moreover, longer-term goals are being considered to expand the mapping system to the rest of the country and to use the data more widely in different public health programmes.

## Conclusions

Housing cartography and household enumeration are fundamental for adequate planning and implementation of household-based public health interventions. The enumeration system presented here is built around an unambiguous coding system and is supported by a software infrastructure that permits updating the household database in real-time. Because the cartographic system itself was developed using a combination of satellite imagery and field enumeration, this methodology is applicable and adaptable to a wide range of settings. The Bioko Island household database represents the backbone of all malaria interventions and, thus, has contributed greatly to the consolidation of a successful malaria control programme. The enumeration system proposed here, however, is far-reaching and can support virtually any household-level public health intervention.

## Data Availability

Data sharing not applicable to this article as it mainly explains methodological approaches to housing and household mapping and limited descriptive analyses of housing and household distribution are presented as illustrative examples of the mapping system.

## Abbreviations

BIMCP: Bioko Island Malaria Control Project
IRS: indoor residual spraying
LLINs: long-lasting insecticide-treated nets
MIS: malaria indicator survey
GPS: global positioning system
GIS: geographical information system
PDA: personal digital assistant
CIMS: Campaign Information Management System
